# Testing novel facial recognition technology to identify dogs during vaccination campaigns

**DOI:** 10.1038/s41598-023-49522-2

**Published:** 2023-12-12

**Authors:** Anna Maria Czupryna, Mike Estepho, Ahmed Lugelo, Machunde Bigambo, Maganga Sambo, Joel Changalucha, Kennedy Selestin Lushasi, Philip Rooyakkers, Katie Hampson, Felix Lankester

**Affiliations:** 1 Boyd Orr Centre for Population and Ecosystem Health, School of Biodiversity, One Health and Veterinary Medicine, University of Glasgow, Glasgow, G12 8QQ UK; 2 Environmental Health and Ecological Sciences Thematic Group, Ifakara Health Institute, P.O. Box 78373, Dar es Salaam, Tanzania; 3 PiP My Pet Technologies, Vancouver, British Colombia Canada; 4 Global Animal Health Tanzania, P.O. Box 1642, Arusha, Tanzania; 5 Department of Veterinary Medicine and Public Health, Sokoine University of Agriculture, P.O. Box 3105, Morogoro, Tanzania; 6 Department of Global Health and Biomedical Sciences, Nelson Mandela African Institute of Science and Technology, Arusha, Tanzania; 7 Paul G. Allen School for Global Health, Washington State University, Pullman, WA 99164 USA

**Keywords:** Biological techniques, Infectious diseases

## Abstract

A lack of methods to identify individual animals can be a barrier to zoonoses control. We developed and field-tested facial recognition technology for a mobile phone application to identify dogs, which we used to assess vaccination coverage against rabies in rural Tanzania. Dogs were vaccinated, registered using the application, and microchipped. During subsequent household visits to validate vaccination, dogs were registered using the application and their vaccination status determined by operators using the application to classify dogs as vaccinated (matched) or unvaccinated (unmatched), with microchips validating classifications. From 534 classified dogs (251 vaccinated, 283 unvaccinated), the application specificity was 98.9% and sensitivity 76.2%, with positive and negative predictive values of 98.4% and 82.8% respectively. The facial recognition algorithm correctly matched 249 (99.2%) vaccinated and microchipped dogs (true positives) and failed to match two (0.8%) vaccinated dogs (false negatives). Operators correctly identified 186 (74.1%) vaccinated dogs (true positives), and 280 (98.9%) unvaccinated dogs (true negatives), but incorrectly classified 58 (23.1%) vaccinated dogs as unmatched (false negatives). Reduced application sensitivity resulted from poor quality photos and light-associated color distortion. With development and operator training, this technology has potential to be a useful tool to identify dogs and support research and intervention programs.

## Introduction

Biometrics, such as fingerprints, have been used in a variety of settings to identify individuals. Facial recognition technology is a type of biometric which uses machine learning algorithms to analyze measurements of faces, such as the distance between the eyes and nose. Facial recognition is extensively used for human identification purposes including security screening at airports and banking automated teller machines^1,2^. Biometrics have been applied to wildlife^3,4^ for demographic studies of species such as red-bellied lemurs, *Eulemur rubriventer*^5^, brown bears, *Ursus arctos*^6^, and giant pandas, *Ailuropoda melanoleuca*^7^*.* Biometrics have also proven useful for ecological studies of animals which do not possess distinguishing facial characteristics such as zebras^8^ or for elusive species such as pumas, *Puma concolor*^9^. In domestic and companion animal settings facial recognition technologies have been used to track the origins of beef cattle^10,11^ and to help identify lost pets^12^ through applications such as Pip My Pet™ (http://www.pipmypet.com/) and PetCo Love Lost™ (https://lost.petcolove.org/about). However, the potential for this technology to be used in disease control programs, such as for rabies vaccinations, remains untapped.

Dog-mediated rabies is a neglected zoonotic disease which kills approximately 60,000 people globally each year^13^ with nearly all cases occurring in Asia and Africa^14^. Despite these losses, systematic and consistent vaccination of domestic dogs is effective at controlling the disease^15,16^, although to achieve herd immunity vaccination coverage needs to be maintained above a critical threshold (approximately 20–40%) below which transmission of the virus can be sustained^17,18^. Because of the importance of maintaining herd immunity to control rabies, vaccination coverage is a key indicator that needs monitoring as part of rabies elimination programs to ensure that a sufficient proportion of the dog population has been reached^18^, and that all communities are effectively targeted^19^.

Typically, coverage assessment methods rely on the identification of vaccinated dogs. To have utility in countries where rabies remains endemic, these methods must be inexpensive, easy to use and accurate. Several methods to identify vaccinated dogs are available, yet they vary in effectiveness, accessibility, and durability^19,20^. For example, vaccination cards, while inexpensive, are easily lost or misplaced and may not identify specific dogs within a household. Temporary collars are inexpensive and simple to use but do not remain in place for extended periods of time. More durable collars are expensive and can be removed. Spray paint to temporarily mark dogs is often used but can be washed or rubbed off and is not reliable during rainy seasons or on darker haired dogs. Ear tags typically require sedation for application, are costly and not widely available. Finally, while microchips are permanent and reliable, their use at scale is costly, and acquiring the chips and scanners in many settings is difficult.

Facial recognition is a promising technology for identifying animals which, if effective, could enable vaccinated dogs to be reliably identified. We developed and field-tested the first mobile phone-based application to identify dogs using facial recognition technology. While the technology has been used in North America to identify pets, this is the first time that the technology has been used in dog-mediated rabies endemic settings. The technology was adapted for use in commonly available smart phones and used as a means of identifying dogs and estimating vaccination coverage achieved during mass dog vaccination campaigns. Our objective was to test the feasibility and performance of the technology adapted for this setting, comparing it against microchips as a gold-standard method for identifying vaccinated dogs. Specifically, we aimed to assess how this technology performed in settings where the majority of dogs are free-roaming, and not routinely restrained and where environmental conditions (e.g. extreme sunlight) may affect photograph quality.

## Methods

We adapted an existing facial recognition technology (designed to assist North American pet owners who have lost their domestic dogs) to assess vaccination coverage of free-roaming domestic dogs in rural villages in Tanzania. We tested the technology as part of an existing dog vaccination intervention which involved registering dogs using the facial recognition application, vaccinating dogs against rabies, collecting post-vaccination validation images from the targeted population, matching these images and analyzing the performance of the technology.

### Application development

The technology PiP My Pet™, used for identifying lost pets in the U.S.A. and Canada, was adapted for use in Tanzania. A description of the search engine structure used in the application engine is given in Supplementary Materials.

The facial recognition algorithm used within the application identifies dog’s faces by compartmentalizing key components of the face (eyes, nose, mouth, ear, etc.) and generates a scoring system (based on component shapes and distances between them) that is used to compare images of dog faces. Ideally, the facial photograph should include the dog’s entire face, including ears, with the eyes and nose facing the camera directly, minimizing other objects such as hands, other dogs, animals, or people in the background. Previously stored images that have the highest number of similar components are returned in a matching interface as possible matches to the face in question. Once a sample of the most likely possible matches are presented, the operator must decide whether any are an actual match for the face in question.

The application was adapted for use in remote Tanzanian communities where network connection is limited. Filters were added to minimize the number of potential matches being returned by the search engine. Filters included ‘sex’ (male, female), ‘color’ (black, brown, tan, white), and ‘village’ name. The color filter included a primary and a secondary color to allow for multi-colored dogs. All dogs enrolled in the study belonged to households in discrete villages, with no movement of dogs between villages, allowing ‘village’ to be included as a filter.

The application underwent several iterative developments. For example, (1) a transparent image of a dog was added to the camera viewfinder to assist aligning dog’s facial features to ensure that images captured both eyes and nose looking directly at the camera; (2) a zoom option was added to facilitate taking pictures from a distance for nervous dogs; (3) a burst image feature was added to enable multiple images to be taken quickly, from which the best was chosen. This improved the operator’s ability to take photographs of dogs that moved a lot; (4) the color filter was adapted to tackle the issues of multi-colored dogs and the subjective assessment of coat color (what appeared as tan to one operator might appear as brown to another). To achieve this, the color filter was made less stringent so that dogs were not rejected if the choice of colors did not exactly match. Rather, dogs with either a primary or secondary color match were included; (5) a field was added to record any scars and permanent abnormalities, such as missing limbs or tail, with the option of a photograph; (6) a barcode scanning option was added to the microchip number field for faster and more accurate recording of the microchip number.

Additionally, the user interface for matching dogs underwent several modifications to improve processing of pictures. Specifically, we set a limit of 25 potential matches to be displayed because during initial testing of the matching process we found that if matches were present they were typically presented in the first five images of dogs and also that operator fatigue increased as the number of presented matches increased. We also added a freeze-frame so that the picture requiring matching was visible to the operator as they continued to scroll down to assess pictures that did not fit in the screen.

### Field testing site

**Figure 1:**
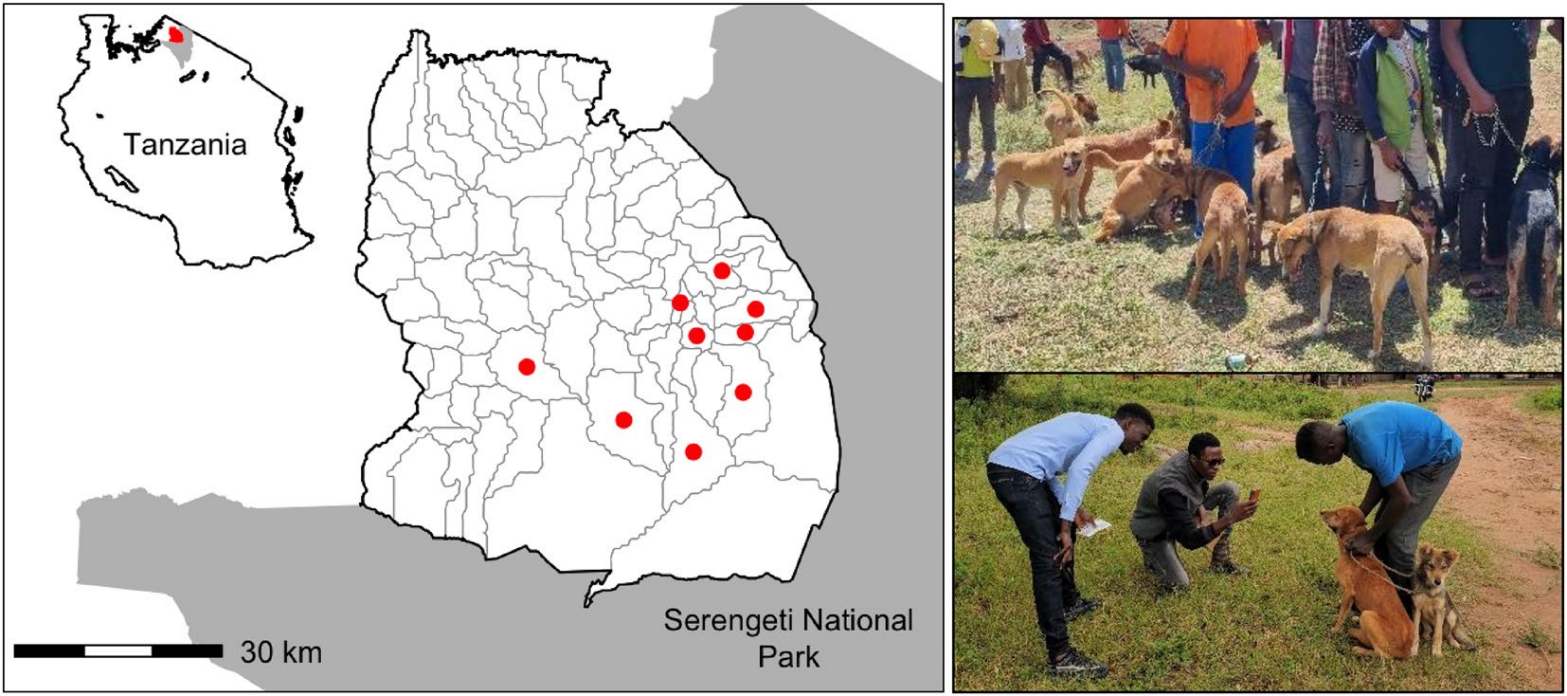
Location of the field testing sites in northwest Tanzania. The central point vaccination locations of the are shown as red dots within the study villages (grey outlines). The Serengeti district is outlined by the black line and the adjacent Serengeti National Park is indicated in grey. The map was generated using R Statistical Software (v4.0.5; R Core Team 2021).

Field testing took place in northern Tanzania in nine agro-pastoral villages within the Serengeti district (population: 249,420 people^21^) where dog ownership is common (human:dog ratio of 6:1–9:1^19,22^) and mass dog vaccination campaigns are conducted annually (Fig. 1).

### Vaccination

In November 2020 vaccination clinics were hosted in nine agropastoral villages in collaboration with the Serengeti District Livestock Office (Fig. 1). Dogs were brought by owners to a central point where eligible dogs were registered using the facial recognition application, microchipped and vaccinated. The study villages are part of a larger ongoing free dog rabies vaccination campaign. Rabies vaccination and microchipping were offered at no cost to the dog owners to prevent socio-economic bias of participating in the study. Dogs were selected for the facial recognition registration if the owner consented to enrolling their dog(s), the dog was at least 8 weeks of age and able to be handled safely and humanely for microchipping and for taking photos of their faces. Dogs that were too nervous or agitated were vaccinated but were not photographed, microchipped and registered in the application. Clinics lasted 1 day in each village.

Registration involved recording in the application the owner’s name and phone number, the dog’s name, sex, reproductive status, and color. Age was selected in months (puppies) or years (adult dogs) from a dropdown menu. Any permanent abnormalities and/or visible scars were noted.

**Figure 2:**
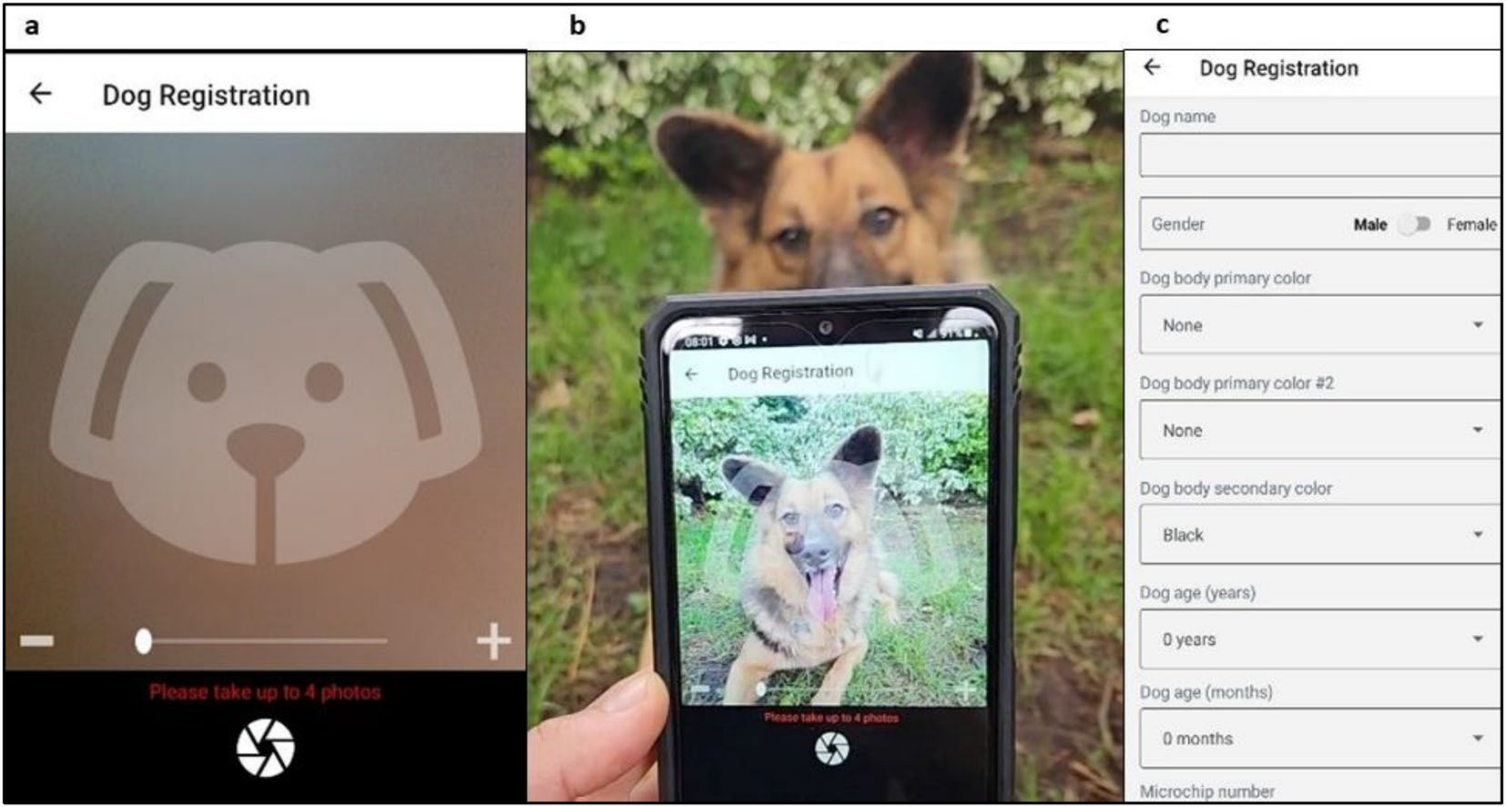
Example of the Facial Recognition application interface. (**a**) Transparent outline of a dog face to guide the placement of the face on the screen; (**b**) dog facing camera; (**c**) application user interface for registering dog information.

Each dog was then photographed aligning the dog’s key attributes, eyes, nose and mouth with the transparent guide on the application (Fig. 2). Ideally the identification picture included both eyes and nose facing the camera directly (both nostrils visible), taking care that the dog's face was not looking down or angled off to the side.

Each dog was microchipped between the shoulder blades, scanned to confirm appropriate placement, and the microchip-specific number entered into the application by scanning the barcode sticker associated with that microchip. The scanned microchip number depicted on the scanner after the dog was microchipped was also photographed and entered into the application for verification purposes. Finally, each dog was vaccinated with the Nobivac™ Canine Rabies vaccine.

Dog owners received a vaccination certificate for each dog vaccinated with a unique registration number generated by the application and the microchip-specific number. At the end of each vaccination day, once connected to internet network, data from the application were uploaded to a server.

### Validation

Validation Day took place the day after vaccination day. An independent research team, which had not been present during the vaccination, visited each village to register as many dogs as possible (whether vaccinated or not). To register dogs, the team walked from house to house enrolling dogs in the study based on the following criteria: (1) the household owner was present and consented to participating (2) the household owned dogs, (3) the dogs were approachable and handleable. Each dog encountered was photographed and registered in the application, scanned for the presence of a microchip and, if present, the microchip number was entered into the application. Thereafter the dog’s details were entered using the same procedure as used on the Vaccination Day. The registered dogs were subsequently used to test the efficacy of the facial recognition technology (see below). Note, because the aim was to register as many dogs as possible to test the application’s efficacy at identifying vaccinated dogs, and because vaccination coverage was not in this instance being assessed, a randomized approach to household selection was not required.

### Matching process to determine which dogs from the validation day had been vaccinated

Matching of dogs was conducted by the Matching Team. To ensure that the operators did not have prior knowledge of which dogs had been vaccinated or not, the Matching Team did not participate in either the Vaccination or Validation Day.

**Figure 3:**
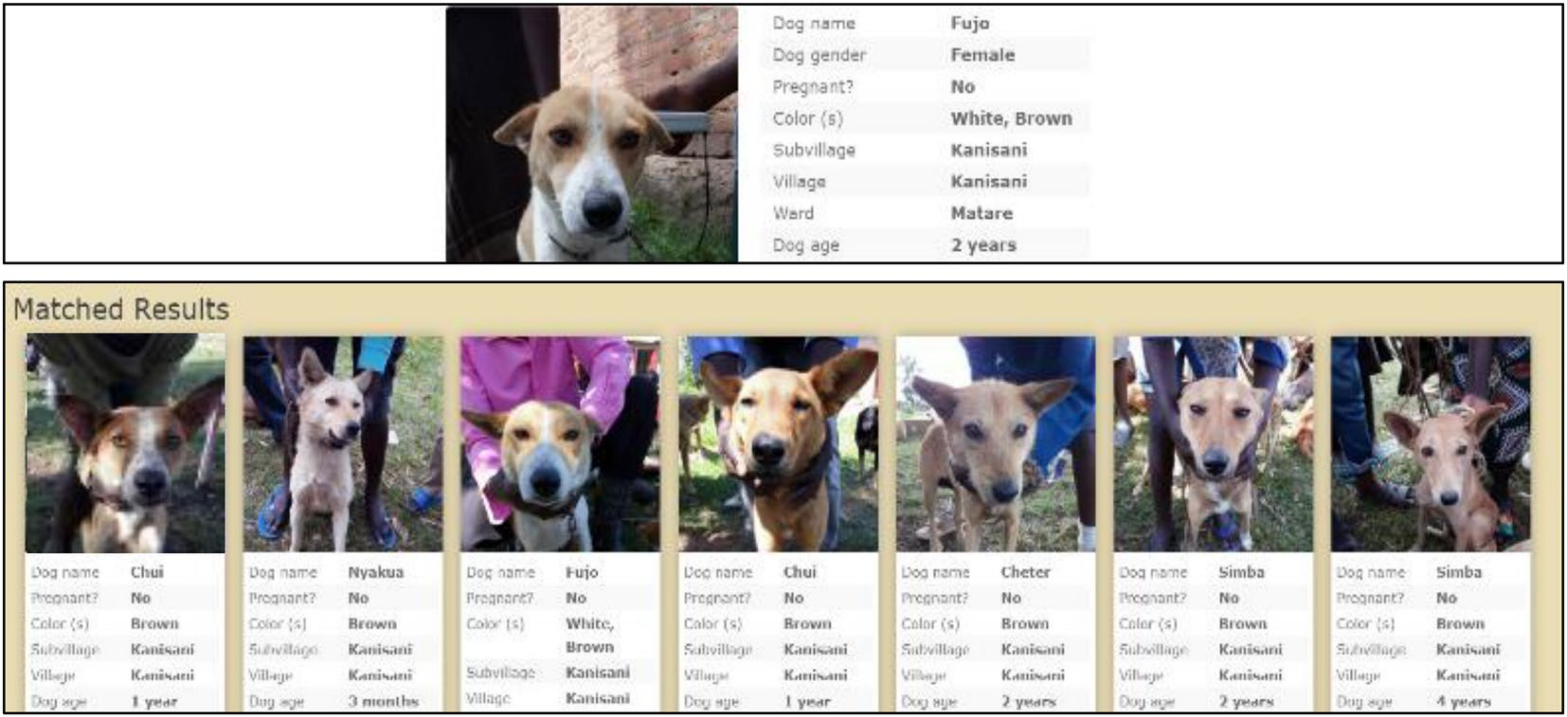
Example of the facial recognition application matching interface. The top picture was registered on the Validation Day. The bottom panel are dogs that were vaccinated and registered during the Vaccination Day and were chosen by the facial recognition engine as possible matches. In this case, the correct match was the third image from the left.

To determine the vaccination status of dogs and assess the performance of the facial recognition technology the following matching process was undertaken:

*Step 1*: Facial Recognition algorithm identifies possible ‘matches’: For every dog registered on the Validation Day, the facial recognition engine generated the closest matches (up to 25) from the image archive of vaccinated dogs that had been taken on the Vaccination Day (Fig. 3).

*Step 2*: Matching team decides: For every dog successfully registered on the Validation Day, the Matching Team decided whether any of the algorithm-suggested matches were an actual match or whether none were. Dogs that were matched were considered vaccinated (matched) while dogs that were not matched were considered unvaccinated (unmatched).

### Validation of the matching process

The matching of dogs was validated using the microchip results which accurately determined whether a dog registered on the Validation Day had been vaccinated or not. Through this, one of five outcomes (further illustrated in Figure S1) were recorded for each dog registered on the Validation Day:

*Outcome 1*: A ‘True Positive’ if the dog was matched by the facial recognition algorithm and had the same microchip number of the vaccinated dog that it was matched with.

*Outcome 2*: A ‘False Positive’ if the dog was matched, however it did not have a microchip.

*Outcome 3*: A ‘False Negative’ if the dog was unmatched but it had a microchip.

*Outcome 4*: A ‘True Negative’ if the dog was neither matched nor microchipped.

*Outcome 5*: A ‘Positive Error’ if the dog was matched and had a microchip however the microchip number was not the same as that recorded for the vaccinated dog that it was matched with.

The compilation of these outcomes across the sample of dogs registered on the Validation Day were used to calculate the sensitivity of the facial recognition algorithm at presenting the correct image within the 25 suggested matches of dogs registered on Vaccination Day (Step 1) and the sensitivity and specificity of the Matching Team at determining whether a dog was vaccinated or not vaccinated (Step 2). These sensitivity and specificity calculations were performed twice. Once on the full set of images and once after the removal of dogs that (a) had their sex recorded incorrectly and (b) where the images were of too poor quality for facial recognition assessment (see Image Screening section below).

### Image screening

The uploaded dog facial images from the Vaccination Day and the Vaccination Validation Day were screened in two steps.

First, the registered sex of the dog from the Vaccination Day was confirmed against the registered sex on the Validation Day. If the sex did not match, for example, if the registered sex on the Vaccination Day was male but on the Validation Day the sex had been recorded as female, or vice versa, these images were excluded from analyses.

**Figure 4:**
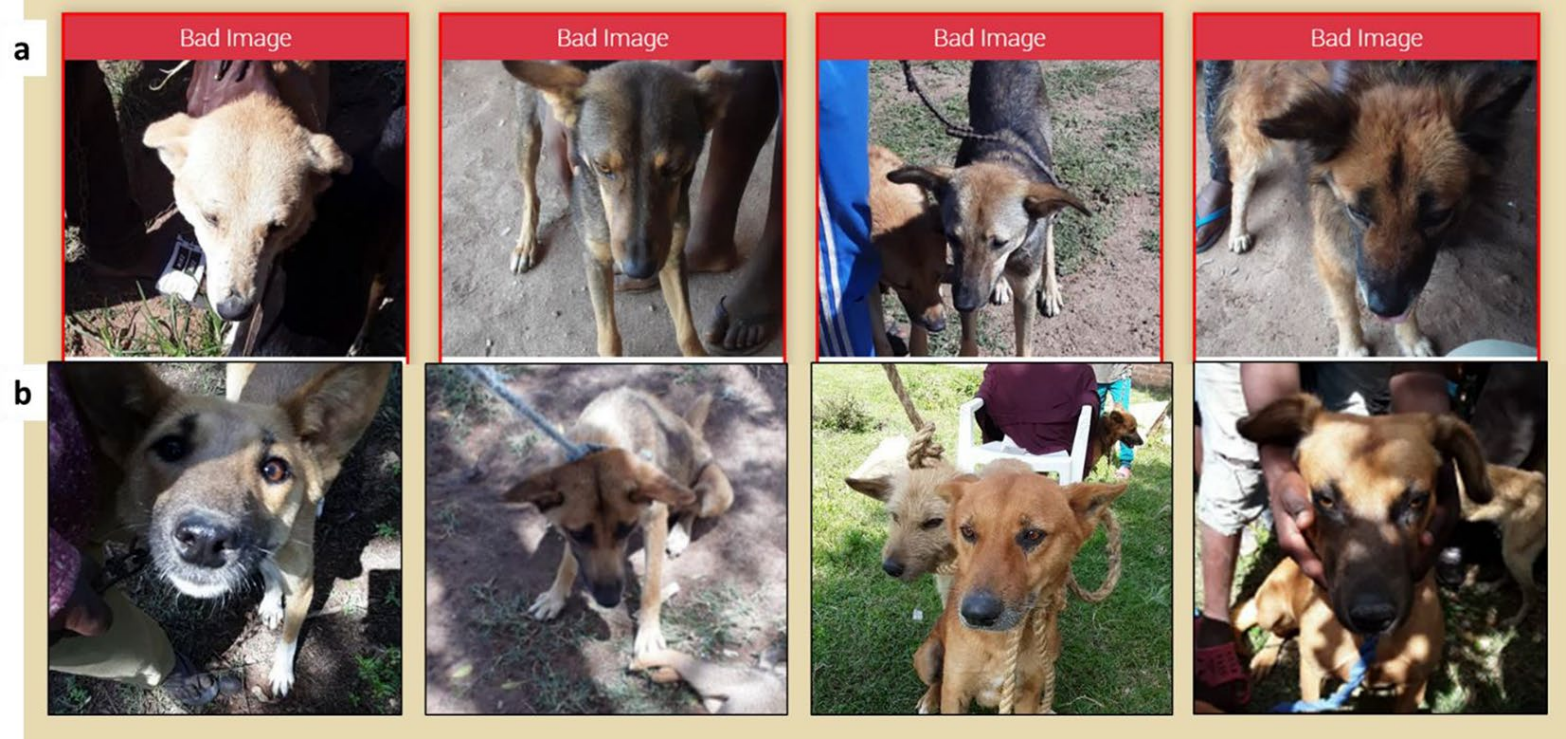
Examples of bad images excluded from the matching dataset. a. Pictures categorized as bad images by the facial recognition algorithm. b. Pictures screened and labeled as bad images by the database administrator. The downward angle of the dogs’ faces, lighting issues, and presence of other faces in the image prevented the facial recognition algorithm from identifying the eyes and nose of the dog.

Second, each image was processed by the search engine and included only if both eyes and nose were identified in the image. For example, an image that was blurred, or with only one eye clearly visible or where the nose was not included were labeled as ‘bad image’ and were excluded. Finally, the remaining images were assessed by a database administrator (who had not been part of either the vaccination or validation teams). Images with multiple dogs or people in the background or where the eyes and nose were not clearly visible were labeled ‘bad image’ by the database administrator and were also excluded (Fig. 4).

### Ethical considerations

This research was reviewed and approved by the Institutional Review Board of Ifakara Health Institute (IHI/IRB/No: 024-2018), and of the Tanzania National Medical Research Institute (NIMRI/HQ/R.8a/Vol.IX/2788). Research was also reviewed and approved by the Tanzania Commission for Science and Technology (COSTECH 2021-471-NA-2019-309) as well as the regional TAMISEMI (Tawala za Mikoa na Serikali za Mitaa) administration for Mara Region. Data collected in the app was encrypted and deidentified. Informed consent for the use of images in an open access publication was obtained from all subjects and dog owners pictured. Participation in the study was strictly voluntary and informed consent was obtained from all dog owners for participation in the study, microchipping, photographing, and registering their dog into the application. This study was performed in accordance with the Declaration of Helsinki and is reported in compliance with ARRIVE guidelines. All methods were performed in accordance with the relevant guidelines and regulations.

## Results

### Vaccination day registration

A total of 3413 dogs across the nine study villages were vaccinated for rabies and recorded in the district livestock register by livestock extension officers. The sex ratio (M:F) of the dogs from the Vaccination Day was 1.62: 1, with 95% of dogs considered adult (4 months and older, n = 3242) and the remaining 5% puppies (3 months and younger, n = 171).

Of these 3413 vaccinated dogs, 1420 (41.6%) dogs were selected for registration into the facial recognition application and were photographed and microchipped. The other 1993 (58.4%) dogs were not included because they either ran away, or the owner did not want to wait, or were too small to microchip (some litters of young pups were vaccinated), or were difficult to handle or photograph. The sex ratio (M:F) of dogs registered into the facial recognition app was therefore 1.95:1, comprising 89.9% adult dogs and 10.1% puppies.

### Validation day registration

On the Validation Day, 720 dogs were restrained, scanned for a microchip, photographed, and registered in the facial recognition application. The sex ratio (M:F) was 1.48:1 of dogs registered on the Validation Day with 83.2% (603) adult dogs and 16.8% (122) puppies. Additional demographic information about the dogs registered in the application can be found in Supplmentary Materials Table S1. 

**Figure 5:**
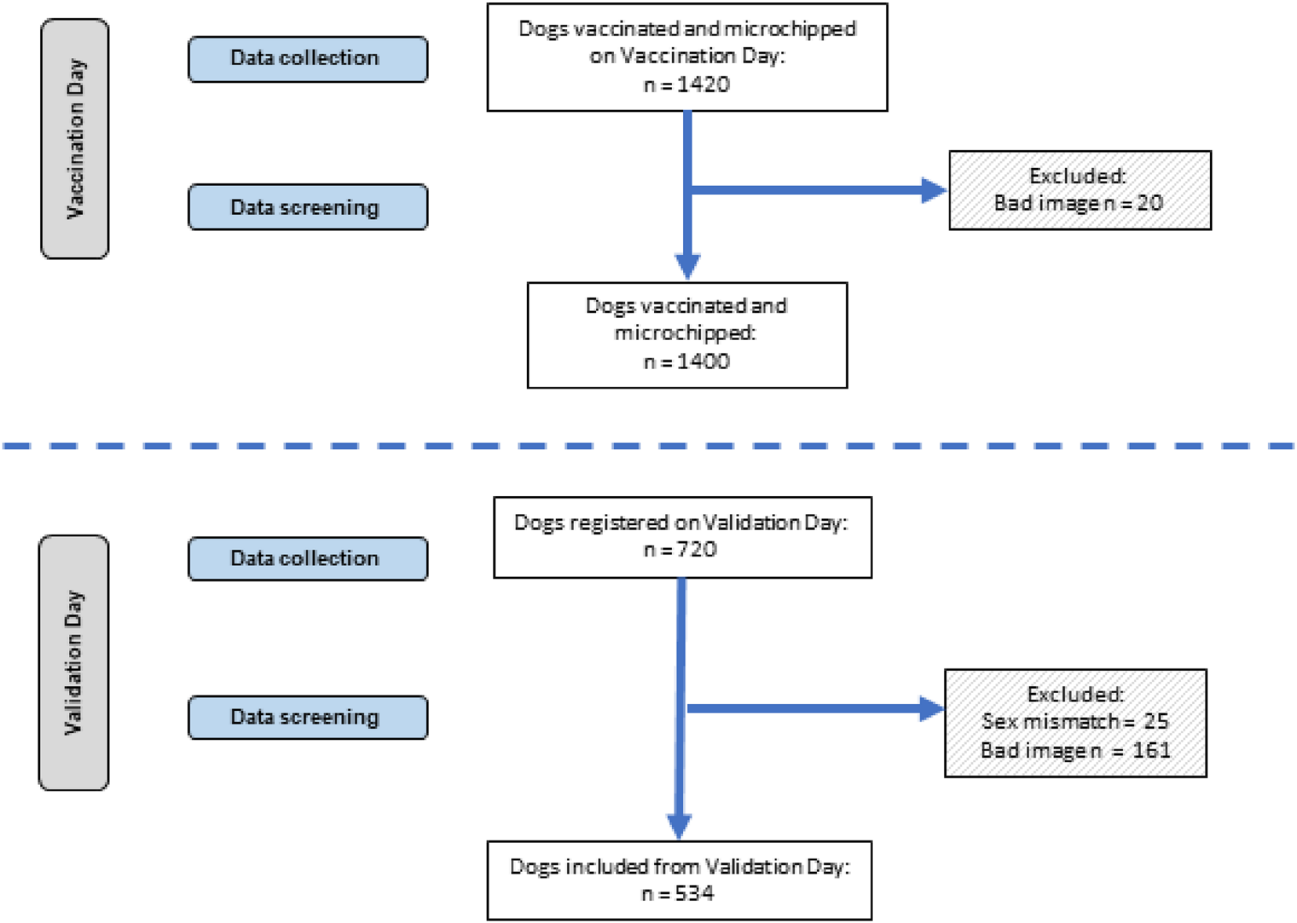
Flow chart of selection procedure for analysis of dog image data. Dog pictures were screened and excluded when: the recorded sex did not match the original registration, images were unclear, and/or the eyes and nose of the dog could not be identified by the facial recognition technology (Fig. 4).

All of the 1420 dogs registered during the Vaccination Day and the 720 dogs registered during the Validation Day were included in the initial matching and calculations of sensitivity and specificity (Fig. 5 and Table 1).

**Table 1:** Matching results of the full dataset.

	Matched correctly	Not matched	Matched to wrong dog	Not attempted	Total
Vaccinated	212 (True positive)	114 (False negative)	10 (Positive error)	24	360
Not Vaccinated	6 (False positive)	354 (True Negative)	–	–	360
Total	218	468	10	24	720

Data shown is for all 720 dogs (without removal of bad images/sex mismatches).

### Image screening: vaccination day

Of the 1420 dogs vaccinated, microchipped, photographed and registered in the facial recognition application (Fig. 5) during the Vaccination Day, 20 (1.0%) were labeled as ‘bad image’s and removed. The remaining 1400 Vaccination Day images were used to match vaccinated dogs that were registered on the Validation Day.

### Image screening: validation day

Of the 720 dogs registered on the Validation Day, 25 (3.5%) had a mismatch in recorded sex (male had been registered on the vaccination day as female or vice versa). From the remaining 695 (96.5%) dogs, 161 (23.2%) were labeled as ‘bad image’ and removed from the analysis. The remaining 534 (74.2%) images were included in the matching process (Fig. 5 and Supplementary Figure S1).

After the screening process, 1400 Vaccination Day images and 534 Validation Day images were included in the matching and sensitivity and specificity calculations (Fig. 5 and Tables 1, 2 and 3).

### Matching process to determine which dogs from validation day had been vaccinated (full dataset)

The first analyses were conducted on the full dataset of all 720 dogs registered on the Validation Day visits. These data included all dogs registered regardless of the accuracy of the sex and image quality (Table 1). Of these 720 dogs, 360 (50%) had microchips and were vaccinated. The remaining 360 dogs (50%) did not have a microchip and were unvaccinated.

*Step 1*: Facial Recognition algorithm identifies possible ‘matches’:

The facial recognition algorithm correctly identified as a possible match 291 (80.8%) of the 360 microchipped and vaccinated dogs (true positive) but failed to identify as a possible match 69 (19.2%) vaccinated dogs (false negative). The facial recognition algorithm sensitivity was therefore 80.8% on the full dataset.

*Step 2*: Matching team decides:

The matching team correctly identified 212 out of 360 vaccinated dogs (65.0%) that had a corresponding microchip (true positive) and 354 out of 360 (98.3%) dogs that had not been vaccinated or chipped (true negative); however, the matching team incorrectly classified as vaccinated 6 (1.67%) dogs that had not been vaccinated (false positive) and classified as unvaccinated 114 out of 360 (31.7%) dogs that had been vaccinated (false negative). Ten dogs (2.8%) were correctly classified as vaccinated but the corresponding dog picture and microchip was not correct (positive error) (Tables 1 and 3). Twenty-four images (3.3%) were not attempted for matching because the registered sex was incorrect. Sex was a hard filter in the application and therefore these dogs did not appear as potential matches for the matching team to choose from.

### Matching process to determine which dogs from validation day had been vaccinated (following image screening process)

After sex mismatches and bad images were removed, the dataset consisted of 534 dogs (Table 2). Of these remaining 534 dogs, 251 (47%) dogs had a microchip and were vaccinated. The remaining 283 (53%) dogs did not have a microchip and were not vaccinated.

**Table 2:** Matching results of the dataset (n = 534) following removal of sex mismatches and bad images.

	Matched	Not Matched	Matched to wrong dog	Total
Vaccinated	186 (True Positive)	58 (False negative)	7 (Positive Error)	251
Not vaccinated	3 (False positive)	280 (True Negative)	–	283
Total	189	338	7	534

**Figure 6:**
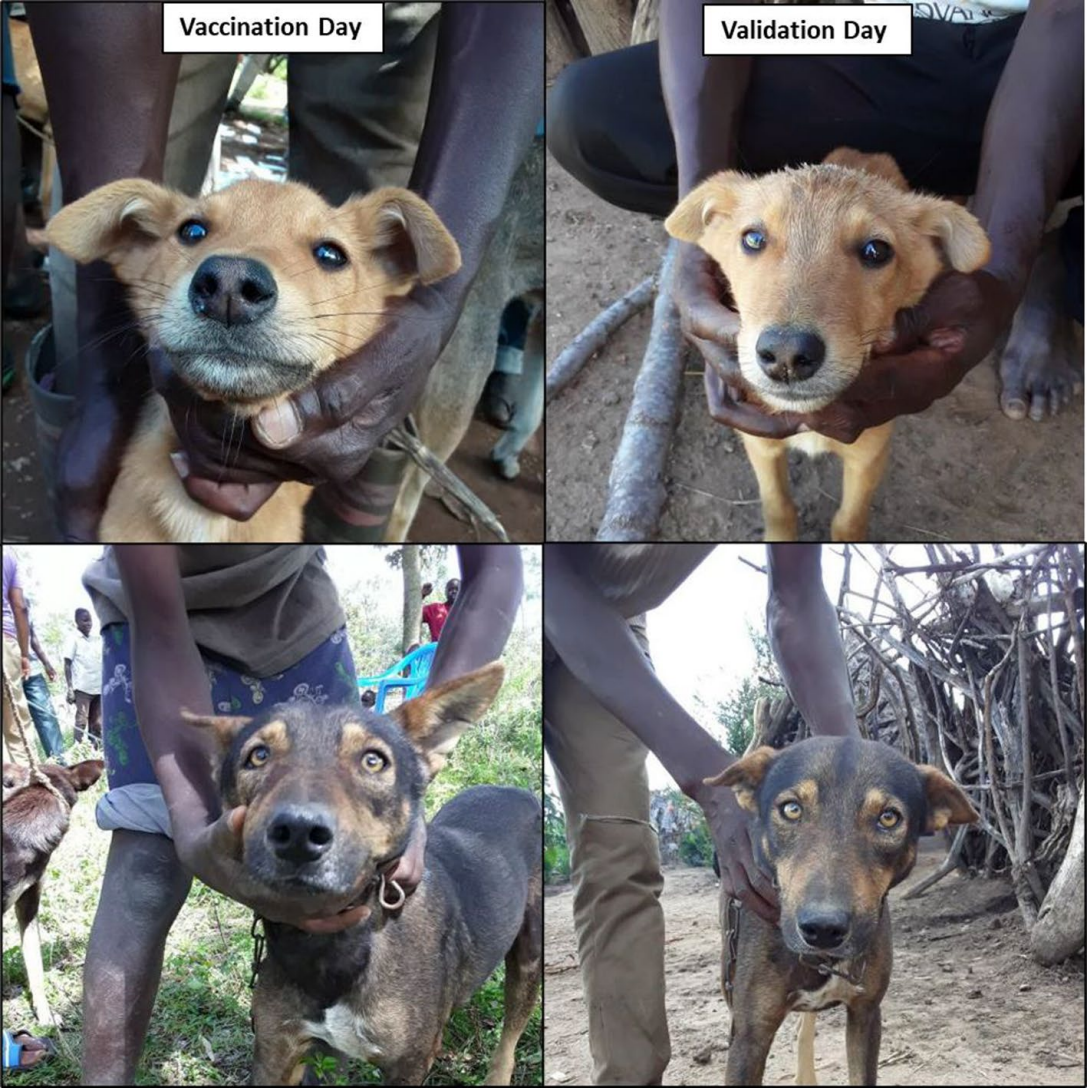
Dog pictures that were not matched by the facial recognition algorithm. The facial recognition algorithm failed to identify a possible match for these two dogs.

Step 1: Facial Recognition algorithm identifies possible ‘matches’:

The facial recognition algorithm correctly identified as a possible match 249 (99.2%) of the 251 microchipped and vaccinated dogs (true positive) but failed to identify as a possible match two (0.8%) vaccinated dogs (false negative). These two dog images were classified as ‘good’ images, however the angle of the dog’s face in the image from the Vaccination Day was very different from the angle of the image from the Validation Day (Fig. 6). The facial recognition algorithm sensitivity was therefore 99.2% on the screened dataset, with sex mismatches and bad images excluded.

Step 2: Matching team decides:

The matching team correctly identified 186 out of 251 vaccinated dogs (74.1%) that had a corresponding microchip (true positive) and 280 out of 283 (98.9%) dogs that had not been vaccinated or chipped (true negative); however, the matching team incorrectly classified as vaccinated 3 out of 283 (1.1%) dogs that had not been vaccinated (false positive) and classified as unvaccinated 58 out of 251 (23.1%) dogs that had been vaccinated (false negative). Seven dogs (1.31%) were matched but the corresponding dog picture was not correct (positive error). The sensitivity and specificity of the facial recognition application increased with the removal of sex mismatches and bad images (Tables 2 and 3).

**Table 3:** Comparison of sensitivity and specificity throughout the data screening process.

Metric	Formula	Facial recognition algorithm full dataset		Facial recognition algorithm following removal of sex mismatches and bad images		Matching team full dataset (%)		Matching team following removal of sex mismatches and bad images (%)	
Sensitivity	TP/TP + FN	291/291 + 69	80.8%	249/249 + 2	99.2%	212/212 + 114	65.0	186/186 + 58	76.2
Specificity	TN/TN + FP	–	–	–	–	354/354 + 6	98.3	280/280 + 3	98.9
PPV	TP/TP + FP	–	–	–	–	212/212 + 6	97.2	186/186 + 3	98.4
NPV	TN/TN + FN	–	–	–	–	354/354 + 114	75.6	280/280 + 58	82.8
Positive Error	PE/Total dogs	–	–	–	–	10/720	1.4	7/534	1.3

PPV, positive predictive value; NPV, negative predictive value; TP, true positive; TN, true negative; FP, false positive; FN, false negative; PE, positive error.

## Discussion

We present results from a field study that aimed to assess the performance of a facial recognition technology developed to identify and differentiate vaccinated dogs from unvaccinated dogs. We demonstrated that, with refinement, facial recognition shows promise as an effective means of identifying individual free-roaming domestic dogs in rural African settings, with potential application to disease control and population management purposes. The facial recognition algorithm had very high sensitivity (99.2%) for correctly identifying vaccinated dogs after screening for bad images and sex mismatches, although the sensitivity of the algorithm was 80.8% on the full unfiltered dataset. The matching team had a lower sensitivity (65.0%) but high specificity (98.3%) for correctly identifying unvaccinated dogs in the full unfiltered dataset, though their sensitivity (76.2%) and specificity (98.9%) improved once bad images and sex mismatches were excluded. Similarly, with the full dataset the positive predictive and negative predictive values of the matching team were 97.2% and 75.6%, respectively. These figures improved to 98.4% and 82.8%, respectively following the exclusion of bad images and sex mismatches. We therefore caution that although the algorithm was accurately able to identify dogs, implementation required training, fine-tuning of the search engine, quality pictures and willing dogs. In its current form, we have shown that the technology can support research purposes requiring identification of individual animals, however, we recommend additional technical improvements and training guidance to increase its potential for programmatic use.

While our study demonstrated that facial recognition technology could be applied to domestic dogs in Tanzania, we identified several limitations that require consideration prior to wide-scale use. Free-roaming dogs in rural Tanzania, as in many low and middle-income countries, are free-roaming and typically are not as sensitized to humans or being handled as frequently as pet dogs in North America or Europe. While all the dogs in our study were owned, most roamed freely and were not accustomed to being handled. To accurately identify and match dogs, the technology requires images with the dogs directly facing the camera so that the distances between the eyes and nose can be triangulated. This was challenging because dogs were not accustomed to sitting still while facing a camera, nor were they used to having their face held by their owners. Additionally, the presence of strangers at their households or large numbers of other dogs and people at vaccination points, made some dogs nervous and excited, and therefore more difficult to restrain to face the camera. Similarly, intense sunlight and lack of available shade in this setting posed challenges for taking good images. When possible, pictures were taken in a shady location however bright sunlight and/or speckling from the shade of tree branches distorted colors and facial features in many images.

Additional challenges in our study included human error and subjectivity. There were several village, sex, and color mismatches in the data set. The sex mismatches occurred typically with people that owned multiple dogs, where two–five dogs were presented at the same time to the vaccination point. While teams did their best to ensure dogs were processed individually in a systematic manner, vaccination clinics were often very busy with more than 200 dogs and their owners waiting in line for vaccination. Consequently, dogs were sometimes mixed up amid registration and resulted in male dogs being registered as female, and vice versa. The color mismatch appeared to be caused by the subjectivity of color description by different people and also colors appearing differently in different light conditions such as in extreme sunlight. For example, in these situations light tan dogs were described as white and brown dogs as tan. Additional training of operators could reduce these errors.

The extra time required to carefully photograph dogs while under restraint sometimes added to the dogs’ anxiety, with some running away before the operator was ready, resulting in missing or unclear photographs. The user’s digital literacy and familiarity with smartphones also influenced the time to photograph and register dogs. With practice we expect processing time and the quality of pictures to improve, nonetheless the increased time taken to process dogs would need to be considered for programmatic use. A further consideration with regards to operator time required, is the matching process with 25 potential matches presented to the operator for review for each registered dog. As the technology is refined and the quality of images improve, the number of potential matches that are presented could be reduced and this will make the matching process less time consuming.

Dog demography is another consideration for this technology. In most free-roaming dog populations, puppies constitute a large proportion of the population; for example, 35% of dogs in rural Tanzania are under the age of 6 months^23^. Our dataset reflected this with puppies 8 weeks–6 months old constituting 27.4% of the dogs registered on the coverage assessment day (Supplementary Table S1). We visited households the day immediately after vaccination therefore minimizing potential changes due to growth. The performance of the search engine on puppies in the weeks or months post-vaccination is not yet known.

Technical considerations for facial recognition technology include mobile devices with high quality cameras, sufficient memory, and network availability. The facial recognition application required a mobile device with a camera with a minimum of five megapixels and sufficient memory (at least 16 GB). Additionally, cameras need to take photographs with a fast trigger time and shutter speed to capture sharp images (highly pixelated images will not match) before a dog moves. Although this application was designed to work offline, a stable network connection is required to upload images for processing and saving. However, once user error, subjectivity and image quality were accounted for, the technology was impressively accurate (98.9% specificity and 76.2% sensitivity) for nondescript mixed breed dogs in this setting (see Fig. 1).

Cost-effectiveness and benefit—cost analyses for this technology were not assessed and will be the subject of future study. In such a study, considerations such as the cost of the equipment (smart phones with quality cameras and large storage capacity), and the processing time required by operators to match registered dogs to determine vaccination status, will need to be quantified and compared against the potential benefits that might accrue. Such benefits could include the ability to permanently identify the vaccination status of dogs, which in turn could lead to vaccine dose savings due to a reduction in the number of times vaccinated (but unmarked) dogs are unnecessarily re-vaccinated. Such analyses and comparisons with existing systems will reveal the utility of the technology.

The use of facial recognition technology for identifying domestic dogs has been focused primarily on pet dogs in North America^12,24^. However, this technology holds promise for health programs in low- and middle-income countries that require dogs to be identified, such as the control of rabies, leishmaniasis, guinea worm and echinococcosis. Presently there are no inexpensive and accurate methods to identify domestic dogs and this can be a barrier for evaluating the effectiveness of disease control programs where it is necessary to identify treated/vaccinated dogs. In this study we demonstrated that facial recognition technology, with some refinement, could be a useful tool for rabies control. Because of the high specificity, and low likelihood of overestimating vaccination coverage, facial recognition could be a supplementary tool to traditional marking methods, such as livestock paint and collars, for evaluating the performance of vaccination programs. Other potential applications include livestock identification for monitoring movement and vaccination for foot-and-mouth disease, lumpy skin disease, peste des petits ruminants, etc^25–27^**.**

### Conclusion and recommendations

In conclusion we find that facial recognition technology, with refinement, can be successfully used on free-roaming village dogs in rural Africa, with application for identifying individuals for research purposes and potential future applications in One Health disease control programs. In our case, we field tested this technology for use in rabies control interventions specifically to estimate vaccination coverage but there are other animal disease and population management purposes for which this technology could prove useful. Technical improvements such as better quality camera lenses and faster shutter speeds and training of users (in animal positioning and handling, light management, for example using an umbrella for shade, as well as user digital literacy) will improve the effectiveness of this exciting technology.

### Supplementary Information

Supplementary Information 1.

Supplementary Tables.

## Data Availability

The dataset generated and analyzed during this study are available in the Supplementary Information.
