# Supplementary material for: Testing novel facial recognition technology to identify dogs during vaccination campaigns
Source: Sci Rep. 2023 Dec 12;13:22025. doi: 10.1038/s41598-023-49522-2 (PMC10716125; doi:10.1038/s41598-023-49522-2)
Supplement: Supplementary file 2 — Supplementary Tables. [file 41598_2023_49522_MOESM2_ESM.docx]

**Supplementary Materials**

**PiP search engine summary**

The search engine used two services to register and search the dog face images. The SimCLR (Simple Contrastive Learning of Visual Representations) model was used to distinguish individual dog faces, and FAISS (Facebook AI Similarity Search) data structure was used to store and search for the dog face images.

SimCLR is a deep learning technique that converts input images into ID (feature vectors) that represents how similar or different the images are compared to other images that were used during training. This search engine uses various dog face images to train the SimCLR model, which provides a model that is able to understand how similar or different individual dog face images are.

Once the SimCLR model provides IDs of different dog face images, a data structure called FAISS is used to store and query them. FAISS provides a feature to easily search from the database for top given number of IDs that are similar to an input ID. For example, if 100 IDs of dog face images are stored that are registered via PIP, and a customer gives us a lost dog face image to search for, FAISS can be used to get top 3 IDs from the database that are similar to the ID of the lost dog face image. Then the mapping between each ID and the dog face image associated to it can be used to load the 3 dog face images that are most similar to lost dog face image that the customer has given.

**Supplementary Table S1. Characteristics of study dogs registered in the facial recognition application during the vaccination day and validation day.**

|  | Sex | Age ≤6 mos | Age ≥7 mos | Total |
| --- | --- | --- | --- | --- |
| Vaccination Day | Male | 191 (20.3%) | 748 (79.7%) | 939 (66.1%) |
|  | Female | 118 (24.5%) | 363 (75.5%) | 481 (33.9%) |
|  | Total | 309 (21.8%) | 1,111 (78.2%) | 1,420 |
| Validation Day | Male | 115 (26.7%) | 315 (73.3%) | 430 (59.7%) |
|  | Female | 82 (28.3%) | 208 (71.7%) | 290 (40.3%) |
|  | Total | 197 (27.4%) | 523 (72.6%) | 720 |

Table S1. Provides the general demographic characteristics of the dogs registered and photographed in the facial recognition application at the Vaccination Day and the Validation Day.

**Supplementary Table S2. Dogs registered in the facial recognition application on the Vaccination Day (see xlsx file FR_Data_SciRep2023 attached).**

**Supplementary Table S3. Dogs registered in the facial recognition application on the Validation Day and their matching data (see xlsx file FR_Data_SciRep2023 attached).**

**Validation Day**

**Data collection**

**Data screening**

**Vaccination Day**

**Data collection**

**Data screening**

Dogs vaccinated and microchipped on Vaccination Day:
n = 1420

Dogs vaccinated and microchipped:
n = 1400

Excluded:

Bad image n = 20

Dogs registered on Validation Day:
n = 720

Dogs included from Validation Day:
n = 534

Excluded:

Sex mismatch n = 25

Bad image n = 161

Microchipped ‘Vaccinated’

n = 360

Not microchipped ‘Unvaccinated’

n = 360

Not matched (False Positive)

n = 20

Matched (True Positive)

n = 212

Not matched (False Negative)

n = 114

Matched (True Negative)

n = 20

Matched to wrong dog (Positive Error)

n = 10

Microchipped ‘Vaccinated’

n = 251

Not microchipped ‘Unvaccinated’

n = 283

Not matched (False Positive)

n = 3

Matched (True Positive)

n = 186

Not matched (False Negative)

n = 58

Matched (True Negative)

n = 280

Matched to wrong dog (Positive Error)

n = 7

**Supplementary Figure S1. Expanded flow chart of study procedures for selection, matching and analysis of dog image data resulting in five recorded outcomes.** Dog pictures were screened and excluded when: the recorded sex did not match the original registration, images were unclear, and/or the dog was not positioned for identification by the facial recognition technology (Fig. 4). More details of the five outcomes are detailed in Tables 1-3.
